# Licoricidin, an Active Compound in the Hexane/Ethanol Extract of *Glycyrrhiza uralensis*, Inhibits Lung Metastasis of 4T1 Murine Mammary Carcinoma Cells

**DOI:** 10.3390/ijms17060934

**Published:** 2016-06-14

**Authors:** So Young Park, Soo Jin Kwon, Soon Sung Lim, Jin-Kyu Kim, Ki Won Lee, Jung Han Yoon Park

**Affiliations:** 1 Department of Food Science and Nutrition, Hallym University, Chuncheon 200-702, Korea; young0122@hallym.ac.kr (S.Y.P.); tnwls2022@naver.com (S.J.K.); limss@hallym.ac.kr (S.S.L.); 2 Advanced Institutes of Convergence Technology, Seoul National University, Suwon 443-270, Gyonggi-do, Korea; kiwon@snu.ac.kr; 3 Biocenter, Gyeonggi Institute of Science & Technology Promotion, Suwon 443-270, Gyonggi-do, Korea; jinkyu90@gstep.re.kr; 4 WCU Biomodulation Major, Department of Agricultural Biotechnology and Center for Food and Bioconvergence, Seoul National University, Seoul 151-921, Korea; 5 Research Institute of Agriculture and Life Sciences, Seoul National University, Seoul 151-742, Korea

**Keywords:** licorice extract, licoricidin, metastasis, breast cancer

## Abstract

Licorice extracts containing glycyrrhizin exhibit anti-carcinogenic properties. Because glycyrrhizin induces severe hypokalemia and hypertension, we prepared a hexane/ethanol extract of *Glycyrrhiza* *uralensis* (HEGU) that lacks glycyrrhizin, and showed that HEGU induces apoptosis and G1 cell cycle arrest and inhibits migration of DU145 human prostate cancer cells. Our previous *in vitro* studies identified two active components in HEGU: isoangustone A, which induces apoptosis and G1 cycle arrest, and licoricidin, which inhibits metastasis. This study examined whether HEGU and licoricidin inhibit metastasis using the 4T1 mammary cancer model. Both HEGU and licoricidin treatment reduced pulmonary metastasis and the expression of CD45, CD31, HIF-1α, iNOS, COX-2, and VEGF-A in tumor tissues. Additionally, a decrease in protein expression of VEGF-R2, VEGF-C, VEGF-R3, and LYVE-1 was noted in tumor tissues of licoricidin-treated mice. Furthermore, the blood concentrations of MMP-9, ICAM-1, VCAM-1, and VEGF-A were decreased in HEGU-treated mice. *In vitro* 4T1 cell culture results showed that both HEGU and licoricidin inhibited cell migration, MMP-9 secretion, and VCAM expression. The present study demonstrates that the licoricidin in HEGU inhibits lung metastasis of 4T1 mammary carcinoma cells, which may be mediated via inhibition of cancer cell migration, tumor angiogenesis, and lymphangiogenesis.

## 1. Introduction

Breast cancer is the most frequently diagnosed cancer among American females and the second most common cause of cancer-related death, behind pulmonary cancer [1]. Like patients with other types of cancer, many breast cancer patients die because tumor cells metastasize to other organs [2]. Therefore, it is important to find ways to prevent and/or delay breast cancer metastasis.

**Figure 3:**
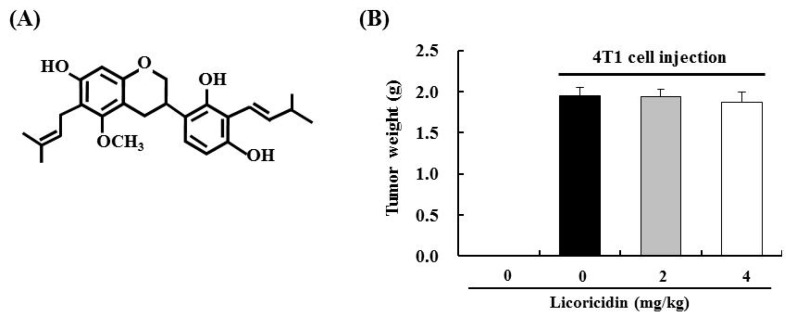

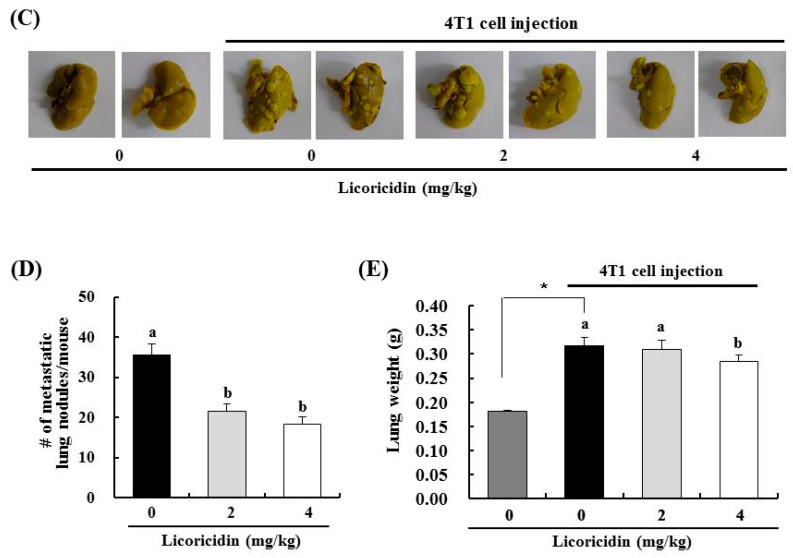
Licoricidin inhibits lung metastasis in BALB/c mice injected with 4T1 cells. (**A**) Chemical structure of licoricidin. 4T1 cells were injected into the inguinal mammary fat pads of female BALB/c mice. For 21 days, beginning on the date of 4T1 cell injection, the mice received intraperitoneal injections of vehicle or licoricidin (2 or 4 mg/kg body weight/day); (**B**) Tumor weight was measured at the time of sacrifice; (**C**) Photographs of the lungs, which are representative of 20 animals; (**D**) The number of tumor nodules; (**E**) Lung weight. Each bar represents the mean ± SEM (normal control, *n* = 8; 4T1 cell-injected, *n* = 20). * Significantly different from the normal control group, *p* < 0.05. Means without a common letter differ among the tumor cell-injected groups, *p* < 0.05.

Humans have been consuming licorice for thousands of years by adding it to food, toothpaste, tobacco, and medicine; licorice exerts many biological effects, including anticancer effects [3,4]. Glycyrrhizin, a triterpene compound, exists in abundance in licorice and has been considered to be an important active component that is involved in the anticancer effects of licorice [3,4]. However, glycyrrhizin has been reported to induce serious hypertension and hypokalemia [5,6]. Therefore, we previously prepared a licorice extract containing an insignificant amount of glycyrrhizin using hexane/ethanol (9:1, *v*:*v*) as a solvent [7]. Our previous *in vitro* cell culture results revealed that the resulting hexane/ethanol extract of *Glycyrrhiza* *uralensis* (HEGU) induced apoptosis [8] and G1 cell cycle arrest [9] and inhibited the metastatic capacity of DU145 prostate cancer cells [10]. Additionally, HEGU supplementation in drinking water inhibited the growth of 4T1 cell allografts in BALB/c mice [9]. We isolated two active components of HEGU: isoangustone A and licoricidin (see Figure 3A for the structure of licoricidin). Isoangustone A induced apoptosis of DU145 human prostate cancer cells [8] and G1 cycle arrest of DU145 cells and 4T1 mammary cancer cells [9], whereas licoricidin inhibited the metastatic capacity of DU145 cells [10]. Other investigators have shown that licoricidin exerts anti-inflammatory [11] and anti-bacterial activities [12].

Metastasis is a multi-step process. In order to complete the metastatic process, cancer cells must migrate out of the primary tumor, intravasate into the lymphatic and/or circulatory systems, evade immune surveillance, survive in the circulatory stream, and enter into and proliferate in remote organs. The net outcome of metastasis is determined by the balance of positive and negative modulators that closely coordinate the action of a variety of molecules, such as extracellular matrix proteinase, cellular junction proteins, and cell adhesion proteins [13,14,15]. The regulation of these molecules may be effective in slowing or halting the metastatic process.

A plentiful body of evidence indicates that both tumor growth and metastasis require angiogenesis. Newly synthesized blood vessels deliver oxygen and nutrients and remove metabolic waste products from rapidly proliferating cancer cells and provide transportation for metastasizing cells (reviewed in [16,17]). Results from studies using transgenic mouse models revealed that angiogenesis occurs early in tumor development, sometimes even prior to tumor formation, and in premalignant stages of cancer [16,17]. As natural components of foodstuffs are generally considered safe for long-term human consumption, such components that can delay and/or prevent angiogenesis should be developed as chemopreventive agents.

Tumor cells are the principal driving force of tumor development and progression. However, in order to proliferate and metastasize, tumor cells need the help of stromal cells derived from bone marrow and normal tissues [18]. Among these stromal cells, immune cells are particularly important for the proliferation and metastasis of cancer [16,17,19,20]. There are many subtypes of immune cells that infiltrate the tumor microenvironment, and the heterotypic interactions between cancer cells and immune cells are quite intricate and dynamic [19]. For example, in malignant tumors, a high number of tumor-associated macrophages (TAMs), especially M2macrophages, are present and drive the ongoing proliferation, angiogenesis, lymphangiogenesis, and metastasis of malignant cells. Consistent with these roles, studies on human tumor samples have revealed that an elevated density of macrophages, especially M2macrophages, is strongly correlated with worse clinical prognoses in many types of malignant cancers. Infiltrating TAMs themselves or the mechanisms through which TAMs differentiate are additional targets for the prevention or treatment of malignant tumors (reviewed in [16,21]).

In the present study, we attempted to evaluate whether HEGU and its active compound licoricidin (Figure 3A) inhibit mammary cancer metastasis using a BALB/c mouse orthotopic model. In this model, 4T1 murine mammary carcinoma cells are injected into the mammary fat pads of immunocompetent, syngeneic BALB/c mice. The patterns of primary tumor growth and metastasis in the 4T1 tumor model are very similar to those associated with human breast cancer [22]. The present results demonstrate that HEGU and its active component licoricidin suppress lung metastasis of mouse mammary 4T1 carcinoma. Licoricidin administration suppressed M2 macrophage accumulation in tumor tissues as well as tumor angiogenesis and lymphangiogenesis. Additionally, licoricidin inhibited cell migration, expression of adhesion molecules, and secretion of matrix metalloproteinase-9 (MMP-9) in 4T1 cells.

## 2. Results

### 2.1. Addition of Glycyrrhiza uralensis (HEGU) to Drinking Water Inhibits Solid Tumor Growth and Decreases Cell Proliferation and the Expression of Proteins Related to Angiogenesis and Inflammation in 4T1 Mammary Tumor Tissues in BALB/c Mice

**Figure 1:**
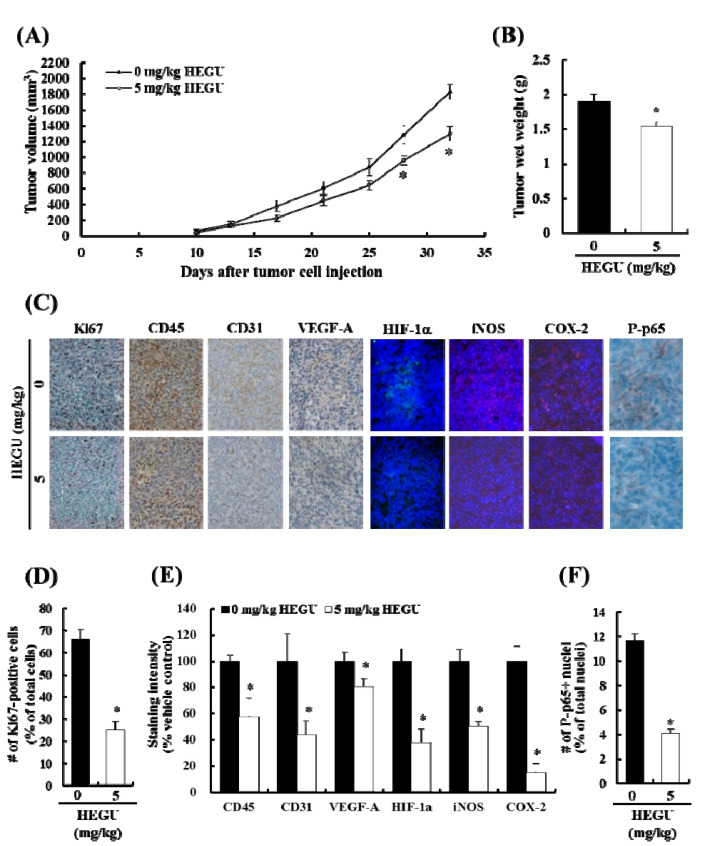
*Glycyrrhiza* *uralensis* (HEGU) in drinking water inhibits solid tumor growth and decreases cell proliferation and the expression of proteins related to angiogenesis and inflammation in 4T1 mammary tumor tissues in BALB/c mice. 4T1 cells (5 × 10^4^ cells) were suspended in 0.1 mL matrigel and injected into the inguinal mammary fat pads of 5-week-old female BALB/c mice. For 32 days, beginning on the date of 4T1 cell injection, the mice were supplied with drinking water containing vehicle or HEGU (5 mg/kg body weight/day). (**A**) Tumor volume was measured using calipers and calculated using the formula (0.52 × long diameter × (short diameter)^2^); (**B**) tumor weight; (**A**,**B**) results are expressed as mean ± SEM (*n* = 20); (**C**) tumor sections were stained with antibodies against Ki67, CD45, CD31, VEGF-A, HIF-1α, iNOS, COX-2, and P-p65 and counterstained with hematoxylin & eosin (H&E) or DAPI; (**D**) The number of Ki67^+^ cells was counted; (**E**) The staining intensity of antibodies against CD45, CD31, VEGF-A, HIF-1α, iNOS, and COX-2 was quantified; (**F**) The number of P-p65^+^ nuclei was counted. (**D**–**F**) Each bar represents the mean ± SEM (*n* = 5). * Significantly different from the tumor control group (0 mg/kg HEGU), *p* < 0.05.

We previously reported that HEGU in drinking water significantly inhibited solid tumor growth of 4T1 cells injected into the mammary fat pads of BALB/c mice [9]. In the present study, we treated 5-week-old female mice with HEGU in drinking water (5 mg/kg bodyweight/day) starting on the day of 4T1 cell injection. Tumor volume was measured with calipers and calculated as (0.52 × long diameter × (short diameter)^2^) [23]. HEGU significantly suppressed tumor growth (Figure 1A,B). The body weight of the mice was not affected by HEGU administration (data not shown).

Immunohistochemical (IHC) and immunofluorescence (IF) staining results revealed that the expression of the cell proliferation marker Ki67 was lower in the tumor tissues of mice fed HEGU than in control mice (Figure 1C,D). The expression of the leukocyte common antigen CD45 was decreased significantly in mice that drank water containing HEGU. The expressions of vascular endothelial growth factor-A (VEGF-A) and CD31 (an endothelial cell marker) were decreased significantly in tumor tissues of mice treated with HEGU. Oral administration of HEGU decreased the expression of hypoxia-inducible factor-1α (HIF-1α) and the HIF-1α target genes inducible nitric oxide synthase (iNOS) and cyclooxygenase-2 (COX-2) [24] in tumor tissues. In addition, HEGU decreased the expression of P-p65NFκB (Figure 1C,F).

### 2.2. HEGU in Drinking Water Inhibits Lung Metastasis in the 4T1 Mouse Tumor Model

**Figure 2:**
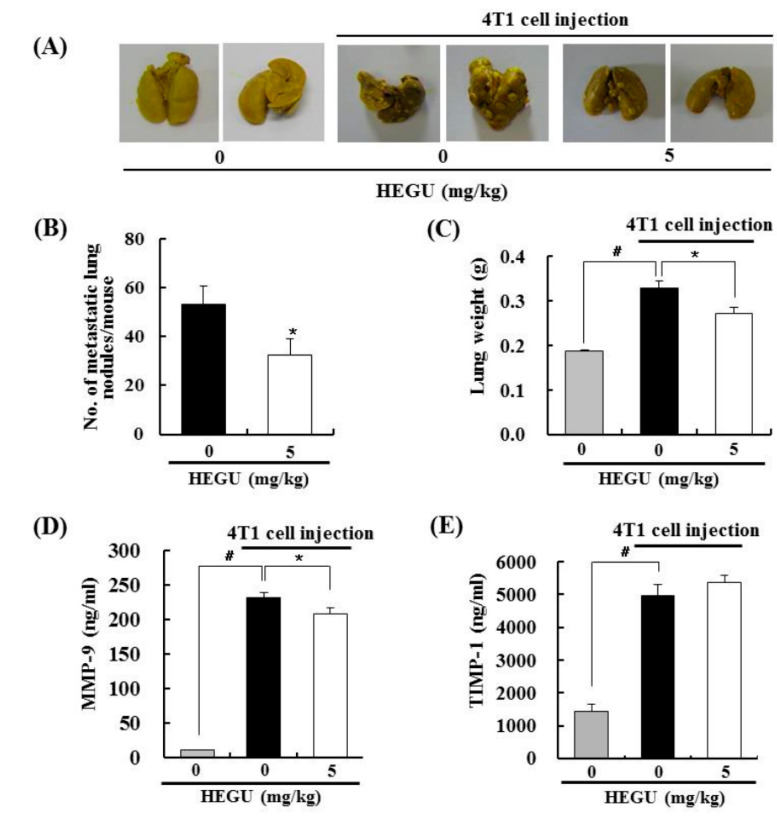

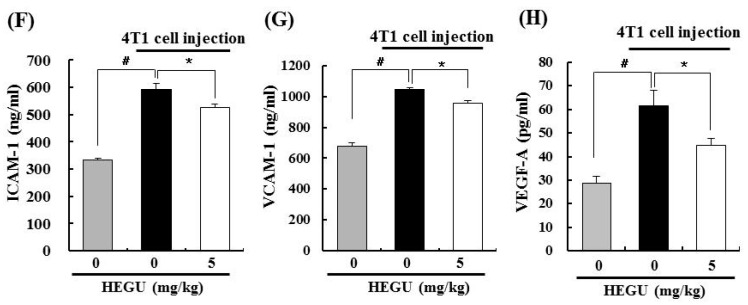
HEGU in drinking water inhibits lung metastasis in BALB/c mice injected with 4T1 cells. Mice were injected with 4T1 cells and treated with HEGU as described in Figure 1. At the end of the experiment, the lungs were excised and fixed in Bouin’s solution. (**A**) Photographs of the lungs, which are representative of 8 normal control animals and 20 tumor-bearing animals. Tumor nodules can be observed on the surface of the lungs; (**B**) the number of tumor nodules in the lungs; (**C**) lung weight; (**D**–**H**) blood samples were collected from the mice at the time of sacrifice, and the sera were prepared. Serum levels of MMP-9, TIMP-1, ICAM-1, VCAM-1, and VEGF-A were measured with ELISA kits. Each bar represents the mean ± SEM (*n* = 8 for normal control, *n* = 19 for 4T1 cell-injected mice). # Significantly different from the normal control group. * Significantly different from the tumor control group (0 mg/kg HEGU), *p* < 0.05.

As our previous *in vitro* results revealed that HEGU inhibits the migration and invasion of DU145 cells [10], we examined the effects of HEGU on lung metastasis of 4T1 mammary cancer cells. The mean number of tumor nodules in the lungs was significantly less in the HEGU group than in the cancer control group (Figure 2A,B). In addition, lung weight in the cancer control group was higher (0.33 ± 0.02 g) than it was in the normal control group (0.19 ± 0.01 g). Lung weight (0.27 ± 0.01 g) in the HEGU-tumor group was lower than that in the cancer control group (Figure 2C). These findings indicate that HEGU not only inhibits the growth of injected tumor cells but also effectively delays spontaneous lung metastasis. To evaluate the possible anti-metastatic mechanisms of HEGU, we examined the effects of HEGU on plasma levels of MMP-9 and adhesion molecules. The injection of 4T1 cells increased the levels of MMP-9, tissue inhibitor of metalloproteinase-1 (TIMP-1), intercellular adhesion molecule-1 (ICAM-1), vascular cell adhesion molecule-1 (VCAM-1), and VEGF-A, and oral administration of HEGU effectively reduced the protein levels of MMP-9, ICAM-1, VCAM-1, and VEGF-A (Figure 2D–H).

### 2.3. Licoricidin Inhibits Lung Metastasis in the 4T1 Mouse Tumor Model

In our previous study, we identified licoricidin as an active component of HEGU that effectively inhibits the metastatic capacity of DU145 cells. Treatment of DU145 cells with licoricidin reduced cell migration and secretion of MMP-9, TIMP-1, urokinase-type plasminogen activator, and VEGF-A [10]. To examine whether licoricidin inhibits mammary tumor metastasis, in this study, 4T1 cells were injected into the inguinal mammary fat pads of female BALB/c mice. For 21 days, beginning on the date of 4T1 cell injection, the mice received intraperitoneal injections of vehicle or licoricidin (2 or 4 mg/kg body weight/day). As shown in Figure 3B, licoricidin did not significantly suppress tumor weight. However, licoricidin significantly reduced the number of metastatic lung nodules (Figure 3C,D). Furthermore, significantly lower lung weight was observed in mice treated with 4 mg/kg licoricidin than in cancer control mice (Figure 3E).

### 2.4. Licoricidin Decreases the Infiltration of Macrophages, Especially M2 Cells, and the Expression of Proteins Related to Inflammation, Angiogenesis, and Lymphangiogenesis in 4T1 Mammary Tumor Tissues in BALB/c Mice

**Figure 4:**
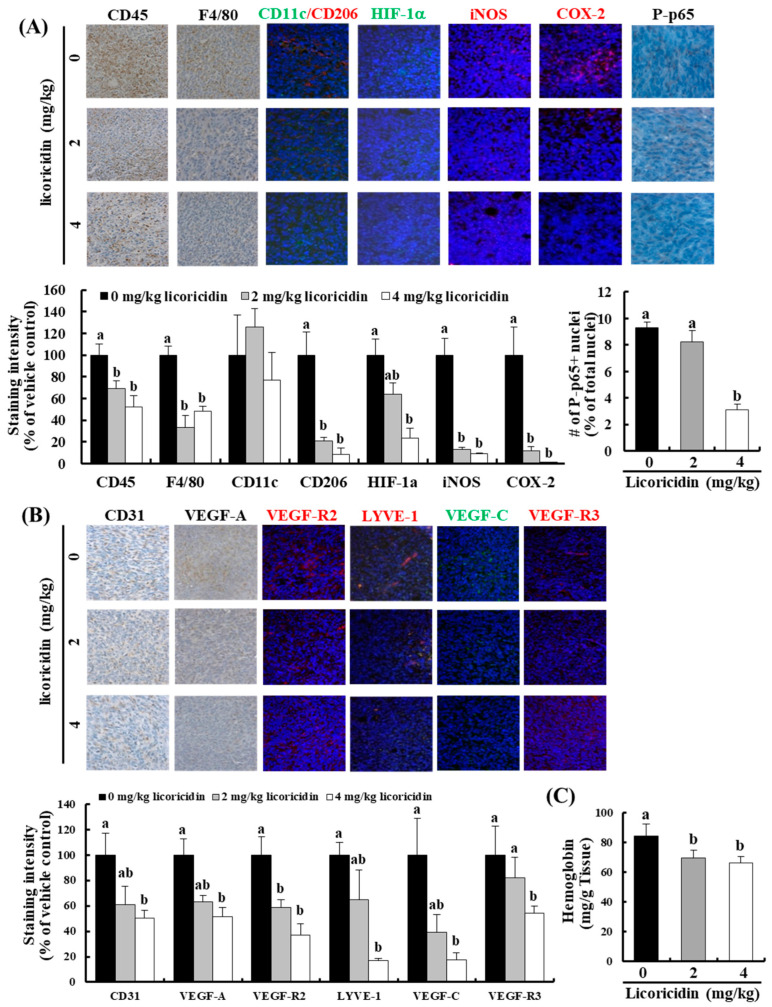
Licoricidin decreases the expression of proteins related to inflammation, angiogenesis, and lymphangiogenesis in 4T1 mammary tumor tissues of BALB/c mice. Tumor sections were stained with antibodies against (**A**) CD45, F4/80, CD206, CD11c, HIF-1α, iNOS, COX-2, and P-p65 or (**B**) CD31, VEGF-A, VEGF-R2, LYVE-1, VEGF-C, and VEGF-R3, and counterstained with H&E or DAPI. Representative immunohistochemical or immunofluorescence images are shown (**upper** panel). Staining intensity was quantified (**lower** panel). Each bar represents the mean ± SEM (*n* = 5); (**C**) Hemoglobin concentrations in tumor tissues. Means without a common letter differ among the tumor cell-injected groups, *p* < 0.05.

As with HEGU treatment, licoricidin treatment decreased the expression of CD45, HIF-1α, COX-2, iNOS, and P-p65NFκB in tumor tissues (Figure 4A). In addition, licoricidin decreased the expression of F4/80 (a mature macrophage maker) and CD206 (an M2 macrophage maker) but did not change the expression level of CD11c (an M1 macrophage maker) (Figure 4A). The expression of proteins related to angiogenesis (CD31, VEGF-A, and VEGF-Receptor(r)2) and lymphangiogenesis (lymphatic vessel endothelial receptor-1 (LYVE-1), VEGF-C, and VEGF-R3) was also decreased significantly in mice that received licoricidin (Figure 4B). The concentration of hemoglobin in tumor tissues was also reduced in mice treated with 2 mg/kg licoricidin (Figure 4C).

### 2.5. HEGU and Licoricidin Inhibit the Migration of 4T1 Cells

**Figure 5:**
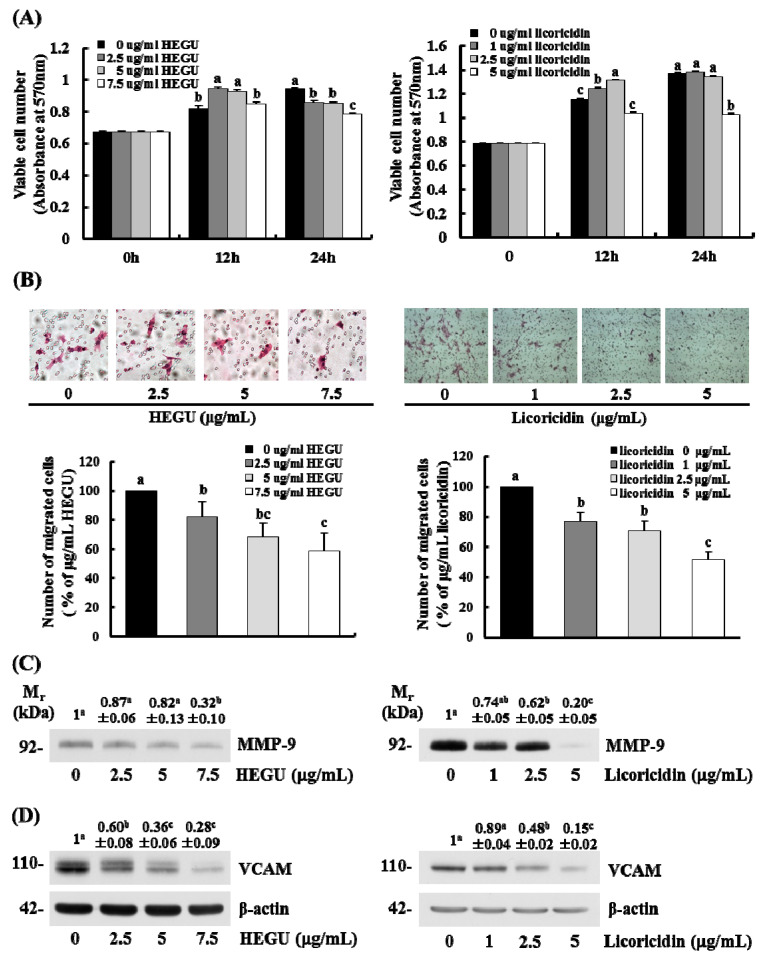
HEGU and licoricidin decrease the migration of 4T1 cells. 4T1 cells were treated as described in Materials and Methods. (**A**) Cell viability was tested using the MTT assay; (**B**) Transwell migration assays were conducted with serum-deprived 4T1 cells. Photographs of H&E stained cells are shown (×100) (**upper** panel). The number of migrated cells were counted and expressed as a percentage of the untreated control cells (**lower** panel). Each bar represents the mean ± SEM of 3 independent experiments; (**C**) 18 h-conditioned media and (**D**) total cell lysates were analyzed by Western blotting. The volume of media loaded onto the gel was adjusted to achieve equivalent protein concentrations. Representative chemiluminescent detection of the immunoblots (from 3 independent experiments). The relative abundance of each band was quantified and the control level was set at 1. The adjusted value (mean ± SEM, *n* = 3) of each band is shown above each lane in the blot. Means without a common letter differ, *p* < 0.05.

**Figure 6:**
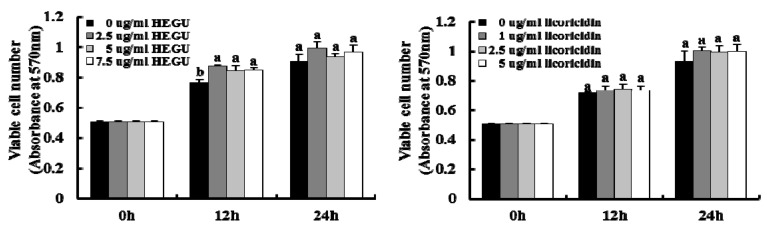
HEGU and licoricidin did not affect the viability of MCF-10A normal mammary epithelial cells. MCF-10A cells were treated as described in Materials and Methods. Cell viability was tested using the MTT assay. Each bar represents the mean ± SEM. Means without a common letter differ, *p* < 0.05.

Because we noted that HEGU and licoricidin inhibited the migration and invasion of DU145 prostate cancer cells in our previous study [10], the present study determined whether HEGU and licoricidin have an effect on the migration of 4T1 mammary cancer cells. As expected, HEGU and licoricidin inhibited the migration of 4T1 cells in a dose-dependent manner (Figure 5B) at concentrations that did not decrease cell viability (Figure 5A). However, cell viability was decreased when these cells were treated for 24 h with 2.5–7.5 g/mL HEGU and 5 g/mL licoricidin. In addition, neither HEGU nor licoricidin decreased the viability of MCF-10A normal mammary epithelial cells (Figure 6). Actually, cell viability was slightly increased when the cells were treated with 2.5–7.5 g/mL HEGU for 12 h. Western blotting results revealed that HEGU treatment (7.5 μg/mL) induced a reduction in the protein levels of MMP-9 (Figure 5C). When 4T1 cells were treated with 2.5–7.5 μg/mL HEGU, the levels of VCAM were significantly lower than they were in cells treated with 0 μg/mL HEGU. Licoricidin treatment (2.5 μg/mL) reduced the levels of MMP-9 and VCAM-1 (Figure 5C).

## 3. Discussion

Licorice and its main component, glycyrrhizin, have repeatedly been shown to have chemopreventive effects. However, chronic intake of licorice containing glycyrrhizin can induce hypertension and hypokalemia [5,6]. Unfortunately, licorice extracts containing high levels of glycyrrhizin are currently widely used as additives in foods and drugs. We previously reported that HEGU lacks glycyrrhizin and contains isoangustone A, and both HEGU and isoangustone A induce G1 cell cycle arrest in *in vitro* cancer cell cultures. We also showed that HEGU suppresses the orthotopic growth of 4T1 allografts and the expression of proliferating nuclear cell antigen and cycle dependent kinase-4 (CDK-4) proteins in tumor tissues [9]. In the present study, using the same mouse tumor model, we demonstrated that supplementation of drinking water with HEGU effectively inhibits the growth of solid tumors and the expression of Ki67, a marker of cellular proliferation [25].

In addition to isoangustone A, our previous study identified licoricidin, a polyphenol, as an antimetastatic agent present in HEGU which can markedly inhibit the metastatic and invasive capacity of malignant prostate cancer cells *in vitro* [10]. In the present study, we demonstrated that HEGU and licoricidin effectively inhibit lung metastasis of mammary cancer cells using the 4T1 mouse tumor model (Figure 2 and Figure 3). Additionally, *in vitro* culture results revealed that HEGU and licoricidin reduced the migration of 4T1 cells (Figure 5). Our animal study revealed that HEGU inhibited the expression of Ki67, CD45, CD31, VEGF-A, HIF-1α, iNOS, and COX-2 in tumor tissues (Figure 1) and plasma levels of VEGF-A, MMP-9, ICAM, and VACM (Figure 2). Licoricidin administration caused a decrease in the expression of proteins related to (1) inflammation (HIF-1α, iNOS, and COX-2); (2) infiltration of macrophages, especially M2macrophages (CD45, F4/80, and CD206); (3) angiogenesis (CD31, VEGF-A, and VEGF-R2); and (4) lymphangiogenesis (LYVE-1, VEGF-C, and VEGF-R3) in tumor tissues (Figure 4A,B). Licoricidin treatment also resulted in a decrease in hemoglobin content in tumor tissues (Figure 4C). These results indicate that licoricidin inhibits migration of cancer cells, infiltration of macrophages, angiogenesis, and lymphangiogenesis, all of which result in a reduction in tumor metastasis. Taken together, these results indicate that HEGU can be developed as a preventive agent to prevent or delay tumor growth and metastasis.

VEGF-A is the key stimulator of angiogenesis in cancerous tissues, in which it is upregulated by oncogene expression, a variety of growth factors, and hypoxia. The formation of new vasculature is critical to tumor formation and metastasis. VEGF-A promotes endothelial cell migration, vascular permeability, and invasiveness, which are required for angiogenesis [26]. Cancers that express VEGF-A are therefore able to grow and spread to other organs. HEGU and licoricidin significantly reduced the expression of CD31 (Figure 1C and Figure 4B), a marker of the vascular endothelium. In addition, licoricidin reduced the expression of VEGF-C, VEGF-R3, and LYVE-1, a specific lymphatic endothelium marker [27], in tumor tissues (Figure 4B). These results indicate that the reduction in tumor angiogenesis and lymphangiogenesis caused by HEGU or licoricidin probably contributed to the reduction in solid tumor growth and lung metastasis that we observed in treated mice.

HIF-1α plays a crucial role in cancer progression by activating the transcription of a broad range of genes involved in the pathophysiology of cancer, including genes implicated in tumor growth, metastasis, angiogenesis, invasion, and metastasis [28,29]. In the present study, the expression of HIF-1α and its downstream targets VEGF-A, COX-2, VEGF-R2, and iNOS [24] were reduced in tumor tissue treated with HEGU or licoricidin (Figure 1C and Figure 4A). In addition, HEGU and licoricidin reduced the expression of phosphorylated p65NFκB. Active NFκB upregulates the expression of genes such as VEGF-C, iNOS, MMP-9, ICAM-1 and VCAM-1that regulate tumor angiogenesis and promote tumor metastasis [30,31]. These results indicate that HEGU and its metastatically active compound licoricidin reduced NFκB and HIF-1α activity, thereby inhibiting the expression of genes involved in the stimulation of tumor angiogenesis.

Tumor cells are commonly surrounded by various stromal cells including fibroblasts, angiogenic vascular cells, and infiltrating immune cells [18]. Of these, infiltrated myeloid cells accumulate in large numbers in avascular and necrotic areas, where local oxygen levels are low, and may support tumor proliferation and progression by promoting angiogenesis and invasion/metastasis [32]. The interactions between malignant cells and stromal cells are complex and dynamic, as some subtypes of immune cells that infiltrate the tumor microenvironment exhibit potent tumor-promoting activity [19,20]. The weight of tumor tissues in HEGU- and licoricidin-treated mice, the infiltration of CD45+ cells, and the expression of COX-2 and iNOS were significantly lower than they were in controls (Figure 1C and Figure 4A). Licoricidin treatment induced a marked reduction in the number of F4/80^+^ and CD206^+^ M2 macrophages. Using the 4T1 tumor model, we previously showed that tumor metastasis was markedly increased when bone marrow-derived M2 macrophages were coinjected with 4T1 cells [33]. Taken together, these results suggest that licoricidin induces changes in the tumor microenvironment, such as a reduction in the number of M2 cells, which contribute to delayed tumor progression.

MMPs are involved in angiogenesis-dependent intravasation and metastasis [34]. In addition, cell adhesion molecules, including ICAM-1 and VCAM-1, have been implicated in tumor progression in cutaneous melanoma [35]. The present results show that the levels of MMP-9, ICAM-1, and VACM-1 were reduced significantly by orally administered HEGU (Figure 2D,F,G). We previously showed that HEGU and licoricidin reduce the invasion, adhesion, and migration of DU145 prostate cancer cells, as well as the secretion of MMP-9, ICAM-1, and VCAM-1 [10]. In the current study, *in vitro* 4T1 cell culture results revealed that HEGU and licoricidin directly inhibit the migration of 4T1 mammary cancer cells as well as the secretion of MMP-9 and VACM-1 (Figure 5C,D). La *et al.* previously reported that licoricidin suppresses the production of cytokines (CCL-5 and IL-6) and MMP-7, -8, and -9 by human monocyte-derived macrophages stimulated with lipolysaccharide, which is associated with reduced activation of p65NFκB [11]. Taken together, the results indicate that the licoricidin present in HEGU has the ability to directly inhibit cancer cell migration, which may be one of the mechanisms by which licoricidin reduces lung metastasis. 

In conclusion, we have demonstrated that HEGU and its active compound licoricidin potently inhibit lung metastasis in BALB/c mice injected with syngeneic 4T1 mammary carcinoma cells. We also confirmed our previous results that showed that HEGU potently inhibits solid tumor growth. The inhibition of tumor angiogenesis and lymphangiogenesis as well as changes in the local microenvironment of tumor tissues may be important mechanisms for the anticarcinogenic effects of HEGU and licoricidin. As HEGU contains isoangustone A, which induces G1 arrest and apoptosis, and licoricidin, which inhibits metastasis, HEGU could prove to be an excellent preventive agent against tumor progression. Future clinical trials are needed to examine the effectiveness of HEGU in the prevention of human breast cancer.

## 4. Materials and Methods

### 4.1. Materials

HEGU [7] and licoricidin [10] were prepared as previously described. Antibodies against VEGF-A, platelet endothelial cell adhesion molecule (PECAM-1, CD31), MMP-9, TIMP-1, ICAM-1, and VCAM-1 were obtained from Santa Cruz Biotechnology (Santa Cruz, CA, USA). Antibodies against Ki67 and HIF-1α were obtained from Abcam (Cambridge, UK). An antibody against mouse CD45 and enzyme-linked immunosorbent assay (ELISA) kits for mouse/rat MMP-9, TIMP-1, ICAM-1, and VCAM-1 were purchased from R&D Systems (Minneapolis, MN, USA). Antibodies to iNOS and COX-2 were obtained from BD Transduction Laboratories (Lexington, KY, USA). If not otherwise specified, all other materials were purchased from Sigma (St. Louis, MO, USA).

### 4.2. Cell Line

4T1 murine mammary carcinoma cells and MCF-10A normal mammary epithelial cells were obtained from the American Type Culture Collection (Rockville, MA, USA). 4T1 cells were cultured in DMEM containing 100 mL/L FBS, 100,000 U/L penicillin, and 100 mg/L streptomycin. MCF-10A cells were cultured in DMEM/F12 containing 50 mL/L horse serum, 0.5 mg/L hydrocortisone, 10 mg/L insulin, 20 μg/L EGF, 0.1 mg/L cholera toxin, 100,000 U/L penicillin, and 100 mg/L streptomycin.

### 4.3. Animal Study Design

Four-week-old female BALB/c mice were purchased from Orient Bio (Seongnam, Korea) and were allowed to acclimate for 1 week under pathogen-free conditions. All animal experiments were approved by the Animal Care and Use Committee of Hallym University (Protocol approval #: Hallym2009-59, 2009-127). The mice were fed an AIN76 diet (Research Diets, New Brunswick, NJ, USA) and given water *ad libitum*. After acclimatization, 4T1 cells (5 × 10^4^ cells suspended in 0.1 mL matrigel (BD Biosciences, San Jose, CA, USA)) were injected into the inguinal mammary fat pads.

To examine the effects of HEGU, 48 mice were divided randomly into three groups: (1) normal control (sham-injected, *n* = 8); (2) 4T1 cell-injected (*n* = 20); and (3) 4T1 cell-injected + 5 mg HEGU/kg body weight/day (*n* = 20). From the date of 4T1 cell injection, the mice were supplied with drinking water containing the vehicle (corn oil) for groups (1) and (2) or HEGU for group (3). Water intake was monitored every day and the concentrations of HEGU in drinking water were modified such that HEGU intake was 5 mg/kg body weight/day.

To examine the effects of licoricidin, 68 mice were divided randomly into four groups: (1) normal control (sham-injected, *n* = 8); (2) 4T1 cell-injected (*n* = 20); (3) 4T1 cell-injected + 2 mg licoricidin/kg body weight/day (*n* = 20); and (4) 4T1 cell-injected + 4 mg licoricidin/kg body weight/day (*n* = 20). One week after 4T1 cell injection, the mice were intraperitoneally injected with saline (+vehicle) or licoricidin (2 or 4 mg/kg body weight/day) for 21 days. Licoricidin was dissolved in DMSO (at 10 mg/mL) and diluted with physiological saline (vehicle).

Tumor volume was measured with calipers and calculated as (0.52 × long diameter × (short diameter)^2^) [23]. Thirty-two days (for HEGU) or twenty-eight days (for licoricidin) after the 4T1 cell injections, the animals were anesthetized via an intraperitoneal injection of 2.5% avertin, and blood was collected from the orbital venous plexus. After blood collection all mice were sacrificed by carbon dioxide asphyxiation and the tumors, lungs, livers, kidneys, and spleens were isolated from the mice and weighed. The lungs were fixed in Bouin’s solution for the quantification of visible metastatic tumor nodules.

### 4.4. Immunohistochemical (IHC) and IFstaining

The tumors were fixed in 4% paraformaldehyde for IHC analysis or frozen for IF staining. IHC and IF analyses were performed as previously described [33]. Photographs were obtained using an AxioImager microscope (Carl Zeiss, Jena, Germany).

### 4.5. Enzyme-Linked Immunosorbent Assay (ELISA)

Plasma was prepared (control, *n* = 8 mice; 4T1-injected, *n* = 19). The levels of MMP-9, TIMP-1, ICAM-1, and VCAM-1 in plasma were measured using ELISA kits.

### 4.6. Western Blot Analysis

4T1 cells (1 × 10^6^ cells/100 mm culture dish) were plated in DMEM containing 100 mL/L FBS. The cells were then serum-starved for 24 h in DMEM and treated with or without various concentrations of HEGU or licoricidin for 18 h. Conditioned media was collected and concentrated as previously described [10]. Proteins in total cell lysates (50 μg protein) [36] and concentrated conditioned media (50 or 80 μg protein) [37] were subjected to Western blot analyses. The signal was detected with a chemiluminescence detection system (Millipore, Billerica, MA, USA). The relative abundance of each band was quantified using the Bio-profile Bio-ID application (Vilber-Lourmat, Marine La Vallee, France) and the control (0 μg/mL HEGU or licoricidin) levels were set to 1.

### 4.7. Transwell Migration Assay

Cells were serum-deprived in DMEM containing 10 mL/L FBS for 24 h. The lower side of a 6.5 mm transwell filter was pre-coated with Type IV collagen. Cells were added to the upper compartment at 25,000 cells/well and treated with various concentrations of HEGU or licoricidin. Stock solutions were prepared by dissolving HEGU or licoricidin in DMSO, and all cells were exposed to DMSO at a final concentration of 1 mL/L. The lower chamber was filled with DMEM with 10 mL/L FBS and 1 mL/L BSA. The cells were incubated for 12 h, and the cells that had migrated were stained with hematoxylin and eosin. The number of migrated cells was quantified and expressed as a percentage of the control cells (0 μg/mL HEGU or licoricidin). Separate cell cultures were prepared to determine the number of viable cells, which was estimated with the MTT assay.

### 4.8. Statistical Analyses

Data are presented as means ± SEMs and analyzed via analysis of variance (ANOVA). Differences among the treatment groups were assessed by Student’s *t*-test or Duncan’s multiple-range test, using the SAS system for Windows v. 9.2. (SAS Institute, Cary, NC, USA).

## Abbreviations

HEGU	Hexane/ethanol extract of *Glycyrrhiza* *uralensis*
CD31	Platelet endothelial cell adhesion molecule-1
VEGF-A	Vascular endothelial growth factor-A
LYVE-1	Lymphatic vessel endothelial receptor-1
HIF-1α	Hypoxia-inducible factor-1α
iNOS	Inducible nitric oxide synthase
COX-2	cyclooxygenase-2
MMP-9	Matrix metalloproteinase-9
TIMP-1	Tissue inhibitor of metalloproteinase-1
ICAM	Intercellular adhesion molecule
VCAM	Vascular cell adhesion molecule
IHC	Immunohistochemical
IF	Immunofluorescence
ELISA	Enzyme-Linked Immunosorbent Assay
VEGF-R	Vascular endothelial growth factor receptor
CDK4	Cyclin-dependent kinase 4
