# Correction: Park et al. Licoricidin, an Active Compound in the Hexane/Ethanol Extract of *Glycyrrhiza uralensis*, Inhibits Lung Metastasis of 4T1 Murine Mammary Carcinoma Cells. *Int. J. Mol. Sci.* 2016, *17*, 934

**DOI:** 10.3390/ijms252011018

**Published:** 2024-10-14

**Authors:** So Young Park, Soo Jin Kwon, Soon Sung Lim, Jin-Kyu Kim, Ki Won Lee, Jung Han Yoon Park

**Affiliations:** 1 Department of Food Science and Nutrition, Hallym University, Chuncheon 200-702, Republic of Korea; young0122@hallym.ac.kr (S.Y.P.); tnwls2022@naver.com (S.J.K.); limss@hallym.ac.kr (S.S.L.); 2 Advanced Institutes of Convergence Technology, Seoul National University, Suwon 443-270, Gyonggi-do, Republic of Korea; kiwon@snu.ac.kr; 3 Biocenter, Gyeonggi Institute of Science & Technology Promotion, Suwon 443-270, Gyonggi-do, Republic of Korea; jinkyu90@gstep.re.kr; 4 WCU Biomodulation Major, Department of Agricultural Biotechnology and Center for Food and Bioconvergence, Seoul National University, Seoul 151-921, Republic of Korea; 5 Research Institute of Agriculture and Life Sciences, Seoul National University, Seoul 151-742, Republic of Korea

In the original publication [1], there was a mistake in Figure 4 as published. In Figure 4A, the images for F4/80 staining were mistakenly duplicated in the slots intended for CD45 staining across the dosages of 0, 2, and 4 mg/kg of licoricidin. We have replaced these images with the correct CD45 staining images for each respective dosage. The corrected Figure 4A appears below. The authors state that the scientific conclusions are unaffected. This correction was approved by the Academic Editor. 

## Figures and Tables

**Figure 4:**
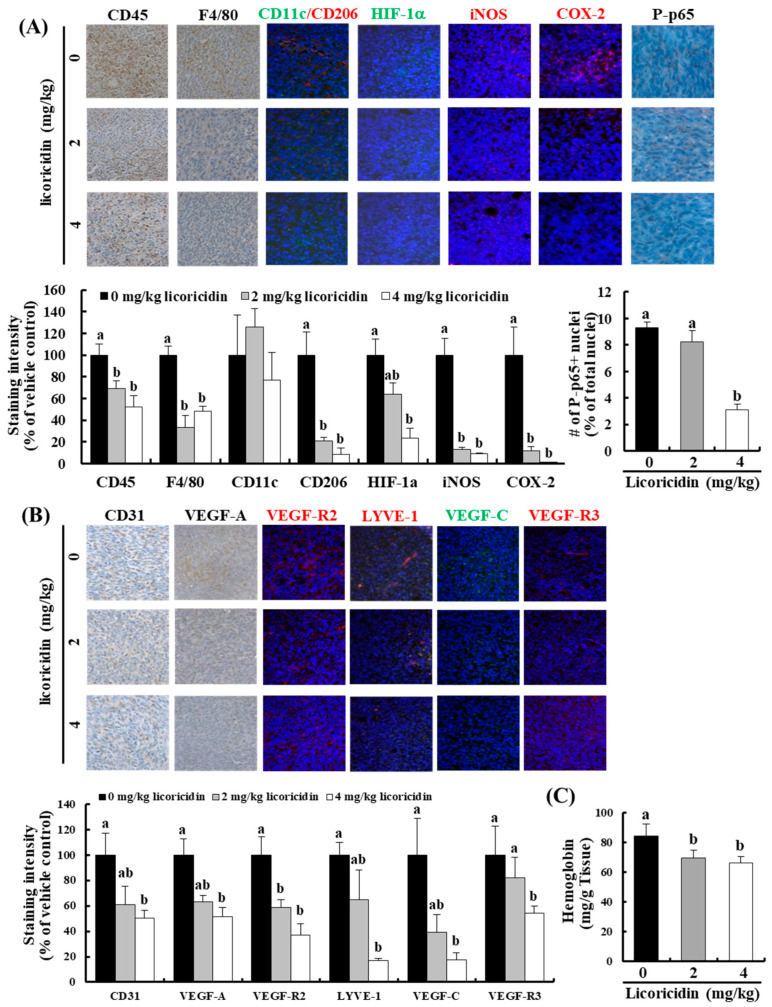
Licoricidin decreases the expression of proteins related to inflammation, angiogenesis, and lymphangiogenesis in 4T1 mammary tumor tissues of BALB/c mice. Tumor sections were stained with antibodies against (**A**) CD45, F4/80, CD206, CD11c, HIF-1α, iNOS, COX-2, and P-p65 or (**B**) CD31, VEGF-A, VEGF-R2, LYVE-1, VEGF-C, and VEGF-R3, and counterstained with H&E or DAPI. Representative immunohistochemical or immunofluorescence images are shown (**upper** panel). Staining intensity was quantified (**lower** panel). Each bar represents the mean ± SEM (*n* = 5); (**C**) Hemoglobin concentrations in tumor tissues. Means without a common letter differ among the tumor cell-injected groups, *p* < 0.05.
